# Non-Pharmacological Interventions for Managing the Symptoms of Depression in Women with Breast Cancer: A Literature Review of Clinical Trials

**DOI:** 10.3390/diseases13030080

**Published:** 2025-03-11

**Authors:** Mayra Alejandra Mafla-España, Omar Cauli

**Affiliations:** 1 Department of Nursing, University of Valencia, 46010 Valencia, Spain; maymaes@alumni.uv.es; 2 Frailty Research Organized Group (FROG), University of Valencia, 46010 Valencia, Spain

**Keywords:** depression, chemotherapy, oestrogens, progesterone, mood, sleep, quality of life, yoga, mindfulness

## Abstract

Symptoms of depression represent a significant burden to patients with breast cancer, not only because of the psychological stress associated with their diagnosis, but also because of the adverse effects of its treatment. We reviewed the clinical trials examining the management of the symptoms of depression in breast cancer patients through different non-pharmacological interventions in different databases (PubMed, Embase, Scopus, and the American Psychological Association). Cognitive behavioural therapy, interpersonal psychotherapy or psychodynamic psychotherapy, as well as acceptance and commitment therapy have been effective in improving symptoms of depression in cancer patients with moderate to severe symptoms. Physical exercise, yoga, mindfulness, and support groups have been shown to benefit patients with mild depressive symptoms. These interventions not only showed positive results in interventions in women with breast cancer in terms of the symptoms of depression, but also highlighted their benefits for comorbid anxiety, stress, and poor sleep quality; moreover, it is suggested that these interventions should be leveraged to manage mental health issues in breast cancer patients. The molecular effects of these interventions, such as the reduction in inflammatory cytokines and cortisol levels, have seldom been reported and need further studies.

## 1. Introduction

Breast cancer (BC) is the most common type of cancer diagnosed in women and one of the main causes of mortality from this disease in this population [1]. Despite advances in cancer treatments and the resulting lower mortality rates, this type of cancer remains a serious public health problem. In part, this is because of the multiple debilitating symptoms that arise because of the surgery, chemotherapy, radiotherapy (RT), and/or biological therapy used to treat this disease [2]. Evidence suggests there is a robust, causal relationship between stressful life events and depression [3]. BC survivors tend to experience an increase in depression symptoms following their diagnosis [4] and often experience adverse physical and psychological symptoms that can persist for years after completing treatment, negatively affecting both their quality of life and health functionality [5,6].

Women who have faced BC report a series of symptoms, either as a direct consequence of the disease or as side effects of the treatment [7]. The psychological symptoms include stress, anxiety, depression, fear of recurrence, and impaired cognitive functioning [8], while the physical symptoms include pain, fatigue, and sleep disorders [8]. Symptoms of depression are common in patients with BC, with the prevalence of clinically significant symptoms reaching up to 46% [9]. These rates are at least double those observed in women in the general population. Indeed, a cohort study of 40,849 Swedish women with BC found that patients with invasive cancer had a 60% higher risk of developing depression and anxiety within 10 years of diagnosis, compared to the general female population [10]. Symptoms of depression are associated with several negative consequences, including an increased risk of suicide, functional impairment, lack of treatment adherence, and prolonged hospital stays [11]. In this sense, a population-based cohort study that assessed the risk of various adverse mental health outcomes in women with a history of BC compared to women who had never had the disease revealed that survival was positively associated with anxiety and depression [12].

Consequently, BC patients face a high risk of depression, highlighting the need for specialised care and a strong support system [13]. Non-pharmacological interventions represent therapeutic approaches that dispense with the use of medications [14]. They are crucial for various reasons, as they not only relieve physical and psychological symptoms, such as anxiety, depression, and stress, but also improve quality of life through the implementation of psychological therapies, such as cognitive behavioural therapy (CBT) [15,16,17], emotional and social support through support groups [18,19], relaxation and mindfulness techniques [20,21,22], supervised physical exercise [23,24], and occupational therapy, among other strategies designed to promote the emotional and psychological well-being of patients [25]. Non-pharmacological interventions are fundamental to a holistic approach in the treatment of depression in women with BC, providing emotional, psychological, and physical benefits that complement and improve the outcomes of conventional medical treatment.

Carrying out this review of the clinical trial literature is justified by the need to update and expand the existing evidence on non-pharmacological interventions for depressive symptoms in women with BC. There is a previous systematic review and meta-analysis on randomised controlled trials (RCTs) that covered studies up to 2017 [26]. The results of this meta-analysis showed that psychotherapy and yoga, or spiritual growth techniques combined with physical activity treatments, are useful in reducing depressive symptoms in women with non-metastatic BC, whether on active or recently completed BC treatment. However, this meta-analysis does not specify the severity of depressive symptoms in that population. Our review covered clinical trials conducted in the last 10 years to the current date and included women with localised or metastatic BC. The women included in the studies were at different stages of the disease: some were in active treatment, others had completed treatment, and some were survivors. Both those with mild to severe depressive symptoms and those with a clinical diagnosis of depression were considered. This update is essential to provide a more complete and updated view on the effectiveness of non-pharmacological interventions in this population. The inclusion of recent studies will allow us to identify new strategies or approaches that could be more effective in the treatment of depression in women with BC, regardless of the severity of symptoms. In addition, this review seeks to answer key research questions, such as this one: What scientific evidence supports the effects of non-pharmacological treatments in reducing depressive symptoms in women with breast cancer? In addition, secondary questions will be explored, such as this one: What is the comparative effectiveness of different non-pharmacological therapies according to the severity of depressive symptoms in these patients?

The findings of this review will be of great relevance to clinical practice, as they will provide updated evidence that will guide health professionals towards the most effective interventions to improve the emotional well-being and quality of life of these patients.

## 2. Materials and Methods

### 2.1. Literature Search

To ensure a comprehensive overview, we conducted a comprehensive literature search that included controlled trials and spanned multiple databases, including PubMed, Embase, Scopus, and those of the American Psychological Association (APA). This search was conducted following the Preferred Reporting Items for Systematic Reviews and Meta-Analyses (PRISMA) guidelines.

To ensure the completeness of the search, we used keywords such as “breast cancer”, “depression symptoms”, “depression”, “hormones”, “chemotherapy”, “psychological symptoms”, and “clinical trials”. The searches covered publications from 2010 to 2024.

### 2.2. Inclusion and Exclusion Criteria

The following inclusion criteria were applied: (1) interventions based on non-pharmacological therapies; (2) assessment of psychological symptoms such as anxiety, depression or stress; (3) study design: clinical trials; (4) language: studies published in English or Spanish; (5) accessibility: studies with full text available.

On the other hand, studies were excluded if (1) they evaluated only pharmacological treatments or surgeries without combination with non-pharmacological therapies; (2) they were case reports, narrative reviews, editorials, abstracts or conference proceedings; (3) they did not present clinically relevant results; (4) they were published in languages other than English or Spanish with no translation available; (5) they did not have access to the full text.

### 2.3. Analysis of Selected Studies

The results of the database search were imported into Mendeley, a web platform used to manage the selection process and eliminate duplicate citations. All identified articles were reviewed in detail, starting with an analysis of their abstract to assess their relevance. In addition to electronic search, a manual search was performed on the references of the retrieved articles to ensure the inclusion of all relevant studies.

Relevant data were extracted and recorded in a previously designed standardised table, which included information such as author, study design, year of publication, country of origin, participants, research objectives, and main findings.

## 3. Results

### 3.1. Study Design and Sample Characteristics

**Figure 1:**
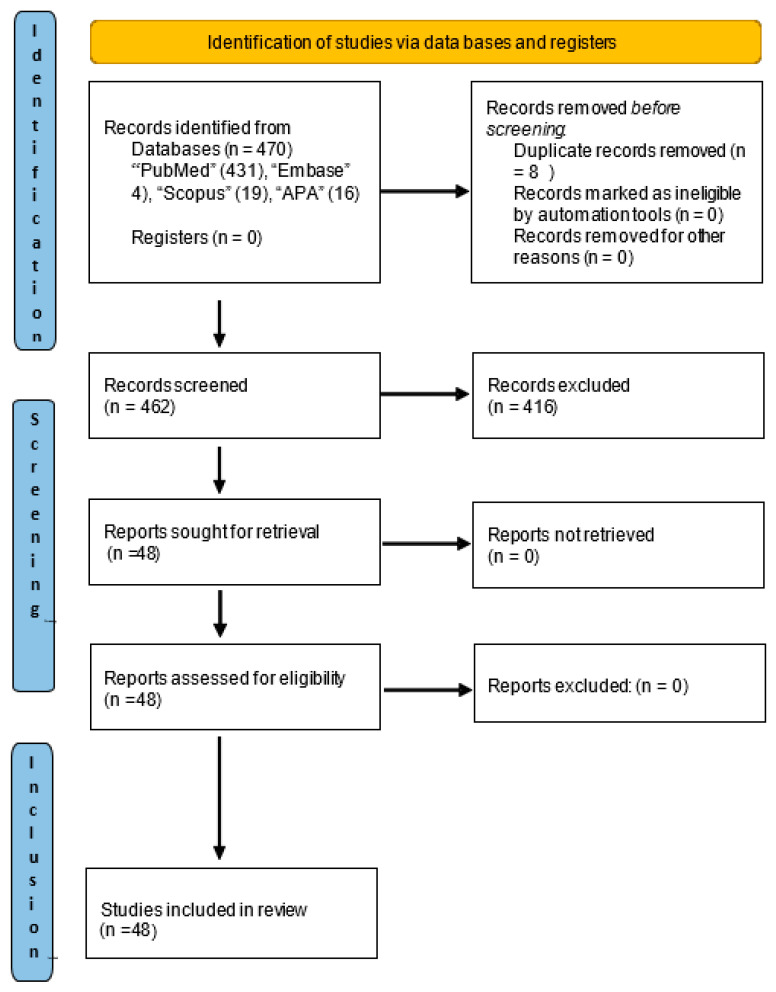
Literature review workflow based on PRISMA statement.

Forty-eight relevant studies were identified, the selection process for which is detailed in the PRISMA flowchart (Figure 1). In addition, a manual search was performed in the reference list of the selected studies, which allowed the identification of two additional articles [20,23]. In total, these studies included 3920 women and assessed the effects of various interventions designed to reduce symptoms of depression in patients with BC (Table 1). Of these, 48 studies were clinical trials with control groups [19,20,23,24,27,28,29,30,31,32,33,34,35,36,37,38,39,40,41,42,43,44,45,46,47,48,49,50,51,52,53,54,55,56,57,58,59,60,61,62,63,64,65,66,67,68,69,70] and had employed a wide range of therapeutic approaches, while 2 studies were classified as observational [71,72]. Two of the latter had carried out comprehensive analyses to monitor the progress of cognitive behavioural interventions [71,72].

Together, these studies represented a comprehensive overview of the current landscape of intervention strategies and observational approaches in the context of BC and depression. The research had been performed on women with localised BC at clinical stages 0 to III [19,20,23,28,29,32,37,38,39,41,42,43,44,48,49,50,53,54,57,59,60,64,66,69,72], stage IV (metastatic) BC [30,31,45,58,62,70], or survivors of stage I–IV BC [27,33,34,36,40,47,51,55,56,63,68,71]. However, some of the articles had not specified the clinical stage of the patients that had participated in the interventions [24,35,46,52,61,65,67]. In these cases, the symptoms of depression were examined in women after having completed their active treatments, which included surgery, RT, and chemotherapy [19,20,23,24,33,34,36,38,40,43,44,46,47,49,51,55,56,57,58,59,60,63,66,68,71,72]. Other studies evaluated these symptoms while women with BC were undergoing active treatment, including RT, chemotherapy, hormone therapy, or concomitant treatments [27,28,31,32,37,39,45,48,52,53,54,61,65,69,70]. The symptoms of depression were also investigated in women scheduled for biopsies [35] or surgeries, such as lumpectomies or mastectomies [29,41,42]. Additionally, some studies had examined the symptoms of depression in women who were not scheduled to receive RT or chemotherapy during the study timeline [64]. Other studies did not specify the type of treatment received [30,62,67].

### 3.2. Interventions Implemented to Alleviate Depression Symptoms in Women with Breast Cancer

In these studies, specific interventions based on cognitive behavioural therapies, mindfulness [20,30,31,36,38,41,42,46,49,51,52,56,57,58,60,65,66,67,68,70,71,72], yoga sessions [32,34,37,39,66], psychological support programmes [33,45,48,50,53,54,55,59,62], and integrated approaches combining various treatment modalities (such as physical exercise programmes [23,24,27,28,40,43,47,63,64], dance therapy [19,61,69], and music therapy [29,35]. These varied methods underscored the importance of tailoring interventions to individual patient needs to provide a comprehensive approach that considers both the physical and emotional aspects of their well-being during BC treatment. In this review, we have summarised the results of specific interventions designed to reduce depression symptoms.

### 3.3. Psychotherapy

In the studies analysed, various interventions had been implemented to alleviate depression symptoms in women with BC. The results revealed that mindful awareness practises (MAPs) reduced the symptoms of depression from pre-intervention to post-intervention compared to the controls [49,57]. Another work evaluated an internet-delivered mindfulness-based cognitive therapy (iMBCT) programme that had resulted in an immediate decrease in depression symptoms compared to the control group [56]. A similar approach, based on stress reduction and mindfulness, also demonstrated a reduction in the symptoms of depression in treated women [58]. Furthermore, an intervention that combined cognitive therapy (CT) and bright light exposure (BLT) showed a greater decrease in depression symptoms compared to the waiting list control group [60].

The application of psychoeducational cognitive behavioural therapy (CBT) also effectively reduced depression symptoms in BC survivors during follow-up [44]. Another work implemented a cognitive behavioural stress management (CBSM) intervention in BC survivors with 5- and 11-year follow-ups [71,72]; women who received the CBSM intervention reported fewer symptoms of depression compared to those in the control group, even up to 15 years later [72]. Another study was based on metacognitive therapy (MCT) and highlighted a decrease in the mean depression score in the intervention group [65]. In turn, the use of acceptance and commitment therapy (ACT) reduced the symptoms of depression compared to the control group [67]. Cognitive behavioural therapy activity stimulation (CBT-AP) showed a significant reduction in the symptoms of depression from baseline to a 3-month follow-up [70]. Furthermore, the implementation of a positive affect skills intervention resulted in reductions in depression and negative affect after 1 month of follow-up [30].

A study based on the Reimagine (symptom self-monitoring) curriculum demonstrated reductions in depression symptoms [36]. In turn, research that implemented mindfulness-based art therapy (MBAT) led to a decrease in depression in the intervention group [38]. Another work implemented a group expression intervention delivered by nurses, which resulted in a decrease in the symptoms of depression compared to the control group [41]. The implementation of a ‘personalised navigation’ intervention by nurses resulted in a long-term decrease in depression levels in the intervention group after 12 months [46]. Work using a mental subtraction meditation (MSM) intervention also reported a decrease in depression [51]. Finally, the REBECCA trial demonstrated that nursing symptom screening and navigation decreased several psychological symptoms, including depression. Hence, all of the findings reported above underscore the effectiveness of a variety of interventions in managing the symptoms of depression in women with BC [52].

In terms of psychotherapy interventions for women with BC and symptoms of depression, a nurse-led programme showed improvements in mood and depression in the intervention group [54]. In turn, a speech therapy programme with psychological and nutritional counselling for 8 weeks resulted in lower depression scores in the intervention group [59]. In a compelling study, women with BC were divided into three conditions: greater self-regulation (ESR), self-regulation (SR), and facts about cancer (CF). In the ESR, the participants wrote about their stressful cancer-related experiences and coping strategies in the first week, deep feelings about BC in the second week, and positive thoughts about the experience in the third week. The instructions were the same for the SR, but the order of weeks 1 and 2 was swapped. Finally, in the CF, the participants wrote objectively about their cancer diagnosis and treatment. Interestingly, the participants in the ESR group showed improvements in the symptoms of depression, while the SR group did not differ from the CF condition. These results suggest that the order of the writing sessions was important and that promoting emotional disclosure before cognitive reappraisal had long-term mental health benefits [33].

Another work implemented supportive care interventions adapted from interpersonal psychotherapy, which resulted in decreased depression scores in the intervention group [55]. However, other researchers found that, although there were improvements in the symptoms of depression both before and after interpersonal psychotherapy (PTI), problem-solving therapy (PST), or brief supportive psychotherapy (BSP), there were no significant differences between these three groups [62]. Similarly, interpersonal psychotherapy implemented in another work also showed improvements in the symptoms of depression in the intervention group [45]. Elsewhere, the use of the short-term psychodynamic psychotherapy (STPP) alleviated depression in the intervention group [48]. In contrast, in two online support group interventions, the usual care (UC) group had lower levels of depression compared to the enhanced prosocial group (P-ISG) [50]. In a psychoeducational intervention compared to a support group, both groups showed improvements over time but no differences in emotional distress were found between them [53].

On the other hand, we found one trial that did not show significant results regarding improvements in depressive symptoms by using the Mindfulness-Based Stress Reduction Program for Breast Cancer (MBSR-BC), the participants in the experimental group showed a tendency to greater improvement in depressive symptoms compared to the control group; however, this difference did not reach statistical significance [20].

### 3.4. Yoga

Work based on a mindfulness yoga intervention together with conventional mindfulness showed that mindfulness yoga was more effective in reducing the symptoms of depression [66]. However, other researchers did not find changes in depression symptoms in the intervention group. Nonetheless, these authors emphasised that the symptoms of depression had increased in the control group during the 8-week waiting time for the intervention and that these symptoms decreased upon its reception, while there were no significant results in the intervention group that participated in yoga sessions [32]. Similarly, a yoga intervention implemented for 8 weeks did not improve depression and anxiety levels in its participants [37]. Moreover, a 12-week yoga intervention produced no changes in patient depression levels [34]. In the same vein, a yoga intervention lasting 6 weeks did not produce improvements in the symptoms of depression [39].

### 3.5. Physical Activity

Work on exercise interventions for women with BC and symptoms of depression was quite varied. Firstly, the administration of 12 supervised social cognitive theory exercise sessions in the BEAT Cancer intervention over 3 months produced improvements in the symptoms of depression up to 3 months later [63]. In turn, changes in physical activity behaviour also reduced depression symptoms from the beginning to the end of another intervention [64]. Similarly, compared to the control group, the symptoms of depression reduced in a 24-week lifestyle intervention that combined three supervised exercise sessions with a healthy eating programme [28].

Another comprehensive study conducted a supervised physical activity intervention using four strategies (supervised exercise sessions, facility access, daily activity promotion, and Fitbit self-monitoring) and applied a full factorial design. These researchers reported a decrease in the symptoms of depression across the entire sample during the 6-month intervention. In turn, the participants who received the active life every day intervention comprising strategies that may positively impact depression symptoms beyond the effect of increased physical activity (i.e., goal setting and social support) experienced even greater symptom reductions [40].

Elsewhere, a Tai-Chi exercise intervention lasting 8 weeks showed lower depression scores compared to the control group [43]. In relation to the aforementioned work, completing a Baduanjin exercise intervention 3 days a week also resulted in improvements in the symptoms of depression because of this specific exercise [47]. However, a Kyusho Jitsu intervention that included martial arts exercises, self-defence, pain cognition, stretching, and physical toning produced no changes in terms of depression symptoms [27].

The study group received a 3-day-a-week aerobic exercise programme for 12 weeks at the gym in 50 min sessions and a home-based resistance exercise programme to be performed at least twice a week for 60 min. This study showed that aerobic and resistance exercise decreased depression levels in women who had previously received breast cancer treatments [24].

On the other hand, in this randomised controlled clinical trial, a home-based exercise programme was implemented for the experimental group, with 20 to 30 min sessions, three times a week for six weeks, starting 72 h after each chemotherapy cycle. However, no statistically significant differences were found in the mean depression score between the two groups, either immediately after the intervention or one month later [23].

The absence of significant results in the reduction in depressive symptoms could be attributed to several factors. First, the duration and intensity of the exercise programme may have been insufficient to generate a significant impact on the participants’ mental health, especially considering that patients undergoing chemotherapy may experience fluctuations in their physical and emotional state.

Furthermore, the timing of the exercises could have influenced the effectiveness of the intervention. Since the sessions were conducted 72 h after each chemotherapy cycle, a period when the side effects of the treatment are typically more intense, it is possible that the participants had difficulty adhering to the programme or that physical discomfort reduced the psychological benefits of exercise. This, in turn, could have limited its impact on reducing depressive symptoms.

### 3.6. Dance Therapy

Regarding dance interventions in these women, in the study that adapted a Chinese ‘square dance’ folk dance, the participants who completed the 16-week intervention reported a lower incidence of the symptoms of depression compared to the controls [69]. Similarly, patients who completed 12 weeks of belly dance classes showed improvements in depression symptoms after the intervention [61]. However, this study did not show significant results in reducing depressive symptoms. The intervention involved dance movement therapy (DMT), with a programme specifically designed for breast cancer patients. This consisted of six 1.5 h sessions, conducted twice a week for three consecutive weeks, throughout radiotherapy treatment. The lack of significant results may be due to the insufficient duration and intensity of the intervention. The dance movement therapy (DMT) programme was conducted for only three weeks, with a total of six sessions. This period may have been too short to generate a significant impact on depressive symptoms. In addition, due to the timing of the intervention: DMT was applied during radiotherapy, a phase of treatment that can generate fatigue, physical discomfort, and emotional stress. These factors could have limited the participants’ ability to fully engage in the intervention, reducing its effectiveness in improving mood [19].

### 3.7. Music Therapy

In the study that combined the use of music therapy and progressive muscle relaxation training in women after having undergone a radical mastectomy, the patients in the intervention group experienced an improvement in their depression levels [29]. Another important study conducted an intervention using either hypnosis or music. In the former, women scheduled to receive a breast biopsy listened to a hypnosis recording that included induction and deepening phases, as well as suggestions to reduce their anxiety and pain and to increase their physical relaxation and emotional well-being; at the end, they were provided with suggestions for self-hypnosis. The music group listened to the same audio, but only its background music and without the suggestions. Interestingly, these authors reported a reduction in depression levels both the intervention groups compared to the control group [35].

Notably, eight studies focused on implementing music therapy interventions in women with BC and symptoms of depression did not change. For example, although the depression scores were reduced in an internet-based cognitive behavioural therapy (iCBT) intervention group, these results were not statistically significant [68]. Surprisingly, depression scores increased in another study that taught a comprehensive educational course on psychological stress and management skills to women with metastatic BC undergoing 5–6 weeks of RT [31]. Additionally, the Swedish Interactive Rehabilitation Information (SIRI) computer-based educational programme did not reduce depression and anxiety levels in its study participants [42].

### 3.8. Effects on the Other Psychological Symptoms: Anxiety, Stress, and Sleep Levels

Many other studies not only showed positive results in terms of depression symptoms but also highlighted the relevance of these interventions in relation to anxiety, stress, and sleep quality. For instance, the MAP-based intervention mentioned above also benefited insomnia symptoms for up to 6 months of follow-up [57]. Additionally, MAPS, another MAP-based intervention, resulted in short-term reductions in perceived stress, sleep disturbances, and increased peace, meaning, and positive affect [49]. The iMBCT-based intervention had long-term effects on anxiety but did not reduce perceived stress or insomnia severity [56]. In contrast, compared to waiting list controls, there were improvements in sleep disturbances in the group receiving GSH-MBI-guided self-help interventions, and these benefits were maintained at 3 month of follow-ups [58]. Lastly, a nurse-led psychological intervention programme also improved anxiety and insomnia symptoms [54].

Other authors demonstrated that patients undergoing CT experienced a reduction in anxiety after treatment [60] and similarly, applied psychoeducational CBT effectively reduced anxiety symptoms during follow-up [44]. The use of MBAT also decreased anxiety level [38]. A group expression intervention delivered by nurses produced a decrease in anxiety symptoms compared to the control group [41], as did a personalised nursing navigation intervention in a long-term follow-up [46]. Finally, a MSM intervention group experienced a reduction in both anxiety and perceived stress [51].

Another study implemented a speech therapy programme with psychological and nutritional counselling that resulted in lower anxiety scores [59], while supportive health education (SHE) interpersonal psychotherapy produced decreased anxiety scores. In the study by Belay et al., there were reduced anxiety symptoms in the intervention group that received interpersonal psychotherapy; these patients were also offered information on sleep hygiene, which helped improve their sleep quality [55]. In a similar vein, a lower incidence of sleep disturbances was reported in the group practising the Chinese folk dance compared to the controls [69]. Furthermore, a music therapy intervention and progressive muscle relaxation training in patients with BC after a radical mastectomy improved anxiety symptoms in the intervention group and resulted in shorter mean hospital stays [29]. Lastly, applied hypnosis reduced stress and anxiety symptoms compared to the control group [35].

Other authors showed that engaging in yoga was strongly associated with fatigue and improved sleep quality but did not impact depression [34]. In turn, implementation of the BEAT Cancer intervention showed improvements in anxiety up to 3 months later [63]. Another study showed that a physical activity behaviour change intervention reduced psychosocial distress in BC survivors [64]. Similarly, the application of a Kyusho Jitsu intervention reduced patient scores on the anxiety subscale from baseline up to 6 months follow-up [27]. Likewise, Tai Chi exercises effectively relieved sleep disorders compared to the control group [43].

Although no significant changes in depressive symptoms were observed between the yoga- and control groups, a notable reduction in inflammation was evident immediately after treatment in the yoga group. Three months after treatment, lower plasma concentrations of IL-6, TNF-α, and IL-1β were still present in the patients from the yoga- compared to the control group. BC patients submitted to a combined intervention based on yoga and stretching displayed a reduction in the cortisol slope at the end of the radiotherapy treatment, the participants in the yoga group showed a significantly steeper cortisol slope compared to the control group [34].

## 4. Discussion

As shown in this review, several non-pharmacological interventions can improve the symptoms of depression in patients with BC. These mainly target physical or psychosocial factors and encompass at least nine different therapeutic strategies, although exercise [23,24,27,28,40,43,47,63,64], psychotherapy [20,33,45,48,50,53,54,55,59,62], and yoga [32,34,37,39,66], alongside meditation, all stand out [30,31,36,38,41,42,46,49,51,52,56,57,58,60,65,66,67,68,70,71,72].

The significant improvements in depressive symptoms observed in these interventions can be attributed to a combination of factors. Stress reduction through psychotherapies such as mindfulness and cognitive behavioural therapy helps patients manage stress and anxiety, which in turn can decrease depressive symptoms [88,89]. Cognitive behavioural therapy has consistently been shown to benefit symptoms of depression. Furthermore, the optimisation of emotional regulation and increased psychological flexibility, facilitated by interpersonal psychotherapy and group therapies, offer a safe space for patients to express their emotions and receive support. The power of positive expectations that patients have, along with social support, provides a sense of belonging and emotional support, which is crucial for mental health and the reduction in depressive symptoms [25]. Traditionally, psychotherapy models were developed and applied in an individual setting, although exceptions exist. The hope of group psychotherapy has always been to provide a similar level of effectiveness. Research suggests that, in fact, group therapy is equally effective as individual therapy [90].

The available evidence on the effectiveness of yoga in reducing symptoms of depression is positive, especially when combined with meditation. However, the effects of exercise on the control of depressive symptoms associated with BC treatment remain inconsistent. These differences between studies may be due to variations in the duration of interventions and other methodological factors. Despite these inconsistencies, a reduction in depression symptoms has been observed with aerobic exercise. However, the potential benefit of exercise may have been underestimated, as clinical trials evaluating this factor also showed benefits in the control group. For ethical reasons, these studies could not restrict exercise due to its known positive effects on physical health. Together, yoga, meditation, and aerobic exercise offer a holistic approach that addresses both the psychological and physical aspects of depression in BC patients. These therapies not only help manage depressive symptoms, but also improve patients’ overall quality of life by promoting physical and mental health [91].

It should be noted that various interventions have been shown to be effective in patients with both moderate and severe depressive symptoms. Many studies recommend the use of cognitive behavioural therapy CBT), behavioural activation, mindfulness-based stress reduction, and interpersonal therapy (IPT) in an individual format. All evidence-based psychotherapeutic treatments for major depressive disorder demonstrated similar and notable improvements in depressive symptoms [62]. Interpersonal therapy (IPT) has been shown to be effective in patients with breast cancer and depression, as it helps to cope with the changes in life and interpersonal relationships associated with the disease. Its approach allows patients to adapt to new role dynamics, express their expectations, and mobilise social support, which contributes to alleviating depressive symptoms [62]. Studies have shown that IPT improves anxiety, depression, and quality of life in women with moderate depression [45]. Furthermore, one study found that twice as many patients with breast cancer and clinical depression achieved remission after receiving short-term psychodynamic psychotherapy compared to the control group (44% vs. 23%) [48]. These findings highlight the crucial role of psychotherapy in emotional management before, during, and after breast cancer treatment. By providing strategies to cope with interpersonal difficulties, fostering self-care, and promoting resilience, psychotherapy helps patients rebuild their identity and regain emotional balance in their recovery process.

This clinical trial evaluated the efficacy of Acceptance and Commitment Therapy (ACT) in women with severe depression due to breast cancer, achieving a reduction in depressive symptoms. The intervention consisted of eight weekly 90 min sessions, promoting acceptance of thoughts and emotions related to the disease, as well as the development of greater psychological flexibility, the main objective of ACT [67].

Likewise, other clinical trials have demonstrated reductions in mild depressive symptoms through various interventions, including: Cognitive Behavioural Therapy (CBT [44], mindfulness practises (MAP) [49,57], mindfulness-based cognitive therapy online mindfulness (iMBCT) [56], face-to-face mindfulness-based interventions (MBI) [58], mindfulness-based art therapy (MBAT) [38], mental subtraction meditation (MSM) [51], combined therapies such as meditation and yoga [66]. Other effective strategies included logotherapy, nutritional counselling [59], physical exercise such as Kyusho Jitsu or Baduanjin [27,47], and the ALED intervention, which combined goal setting and social support, showing benefits in reducing depression beyond the impact of physical exercise [40].

Furthermore, clinical trials that conducted online psychosocial interventions, such as telephone interpersonal intervention (TIPC) and supportive health education (SHE) [54], as well as the LILAC positive affect skills intervention, with sessions both in person and online [30]. The “Reimagine” programme included activities such as attending group meetings and completing cognitive reframing and mind–body exercises [36]. The results suggest that interventions delivered online or face-to-face were effective in reducing mild depressive symptoms.

Clinical trials demonstrated the efficacy of support groups in reducing mild depressive symptoms. A standard support group (S-ISG), focused on information sharing, emotional support, and positive change skill development, was compared to an enhanced prosocial support group (P-ISG), which included advice on how to support others online. Reductions in depression score were observed in the S-ISG group [50]. In addition, the study evaluated psychoeducational group interventions (PEG), focusing on health education, stress management, and problem solving, compared to support groups (SG), which offered emotional support and reduced isolation. Both approaches showed improvements in anxiety and depression over time [53].

Interventions for mild, moderate, and severe depression show similar results, with approaches such as CBT, mindfulness, and meditation having been shown to be effective at all levels. These therapies share the goal of modifying negative thought patterns and strengthening coping skills, promoting positive changes in emotional management [92]. However, the intensity and combination of techniques vary depending on the severity of symptoms. For mild depression, interventions are usually less intensive, focusing on meditation and mindfulness. In contrast, moderate to severe cases require more structured strategies, such as advanced CBT techniques, combined with other therapies and even pharmacological treatment when necessary.

But there are also differences in the results of these interventions on mild, moderate, and severe depressive symptoms. It could be due to the degree of intervention. Moderate and severe depressive symptoms often require more intensive and structured interventions, such as ACT and IPT, which offer a more in-depth approach to addressing emotional and functional crises. In contrast, mild depressive symptoms can be managed effectively with more general, accessible, and less intensive interventions such as stress-reduction meditation, mindfulness-based art therapy, physical exercise, and support groups, which are effective, but less specific. Due to the duration and frequency of the therapies. Therapies for moderate and severe depression, such as CBT and IPT, often involve longer duration and frequency of sessions. For mild depressive symptoms, interventions may be of shorter duration and frequency, better suited to less intensive needs and providing positive results with a smaller investment of time.

The impact of other interventions on the symptoms of depression are also unclear, partly because of the wide range of therapeutic strategies considered, which made it very hard to compare them or estimate a global effect. Therefore, we instead grouped the studies together, allowing us to consider their dimensions and characterise them in more homogeneous groups, according to the strategies they had implemented. Another factor that made it difficult to globally evaluate the impact of these interventions was the range in their implementation formats: from several times a week up to several months. Thus, further work to compare the intensity and duration of the interventions will be necessary to elucidate the minimum requirements for such interventions to produce beneficial effects.

In psychotherapy clinical trials, both the placebo effect and the placebo response may occur. The placebo effect is a psychobiological phenomenon in which the patient’s improvement is due to factors such as his or her positive expectations and belief in the efficacy of the treatment. The placebo response, in contrast, encompasses all changes in the patient’s health following an inactive treatment, including variations in symptoms before and after the intervention [93,94]. In psychotherapy, the placebo response can be influenced by environmental and psychosocial factors, such as the therapeutic relationship, the therapist’s support, and the setting in which the therapy takes place. These elements, supported by empirical evidence, play a key role in therapeutic outcomes [95,96,97,98].

The placebo effect is widely recognised in biomedical research and medical practice, although its neurobiology and neuropsychology are still limited in understanding [99]. However, recent research in areas such as pain, depression, and Parkinson’s disease has shed light on some of the neurobiological mechanisms of the placebo effect. It has been observed that both conscious expectation and unconscious behavioural conditioning processes are fundamental in this phenomenon. These processes can trigger the release of endogenous neurotransmitters such as dopamine, serotonin, and endorphins, which are related to well-being and can mimic expected or conditioned pharmacological effects, i.e., the placebo effect [99]. Although the placebo effect has traditionally been considered to operate primarily on a psychological level, more recent research has revealed that it can also trigger physiological responses in the body. Belief in the effectiveness of treatment can activate areas of the brain associated with emotional regulation and well-being, such as the prefrontal cortex, which could contribute to the reduction in depression symptoms and improvement of mood [100]. In anxiety and depression, placebo responses are associated with increased activity in neural networks related to emotional regulation [101,102]. The main neural pathways through which expectations and memories could influence peripheral immune functions include the neocortical-sympathetic-immune axis, which encompasses limbic and hypothalamic relays; the hypothalamic–pituitary–adrenal immune axis and the brainstem–vagus–cholinergic pathway. Therefore, placebo effects may benefit end-organ functioning and the overall health of the individual through the healing power of beliefs, positive expectations, and conditioning processes [99].

Diagnosing depression in BC patients involves a comprehensive approach that considers psychological and somatic symptoms. Mild to moderate depressive symptoms can be difficult to differentiate from somatic symptoms of BC for several reasons. First, there is significant overlap in symptoms: fatigue, loss of appetite, and insomnia are common in both depression and cancer and their treatments [103,104]. These shared symptoms may make it difficult to accurately identify depression in BC patients. Additionally, the natural emotional reaction to receiving a cancer diagnosis and undergoing aggressive treatments can trigger a variety of emotions, such as sadness, anxiety, and worry. These emotions are normal and expected responses to such a stressful situation and do not necessarily indicate the presence of clinical depression. However, this emotional reaction can mask or complicate the diagnosis of depression [105]. Changes in quality of life due to side effects of cancer treatment may contribute to depressive symptoms, making them difficult to distinguish [24]. Additionally, stigma and denial, where some patients minimise or deny their emotional symptoms due to the stigma associated with depression or the perception that expressing negative emotions is a sign of weakness, may prevent patients from seeking or accepting help for their emotional problems, which in turn complicates the proper diagnosis and treatment of depression [106].

These reasons underscore the importance of a comprehensive and careful assessment by healthcare professionals to differentiate between depressive symptoms and somatic symptoms of cancer. It is crucial that professionals use multidisciplinary approaches that consider both the psychological and somatic aspects of the patient, and that they maintain open and non-judgmental communication with patients to ensure accurate diagnosis and treatment. A proper diagnosis of depression is of vital importance as it allows health professionals to implement effective non-pharmacological interventions, such as psychotherapy, cognitive behavioural therapy, emotional and social support, and other interventions discussed in this literature review. These interventions have been shown to be effective in reducing depressive symptoms, regardless of their severity. These interventions have shown that they can significantly improve the emotional well-being and quality of life of BC patients who present with depressive symptoms.

### Limitations of the Review

This review has certain limitations that should be taken into account when interpreting and generalising the results. Firstly, the search for articles was conducted in English and Spanish, which might have excluded relevant studies published in other languages.

Secondly, the methodological heterogeneity of the selected clinical trials and interventions analysed made it difficult to compare the results of the studies included in this review. The heterogeneity in the interventions and outcome variables, the differences in the populations as within the BC there were women in the studies who had previously undergone chemotherapy, concomitant hormonal therapy or only undergone surgery and the wide age range of the patients may be a difficulty in generalising the benefits of each type of intervention. The lack of quality analysis of these clinical trials due to different experimental design, e.g., with or without control group, with or without randomisation, is a limitation when analysing their effects. Each type of intervention has different mechanisms of action, which complicates direct comparison and uniform assessment of their efficacy in addressing the improvement of depressive symptoms. This challenge is further compounded by the cultural heterogeneity of the participating population, which is explained in more detail in the next section. To reduce this heterogeneity, we recommend using guidelines such as PRISMA or CONSORT in future research to improve methodological consistency.

Third, each study had its own criteria for measuring the efficacy of the interventions, as well as different inclusion criteria, so it is possible that the studies did not take into account the possibility of confounding variables. Furthermore, the use of different scales to measure depressive symptoms introduces variability in the results, which may complicate comparison between studies and compromise the overall conclusions.

## 5. Conclusions

This review highlights the importance of considering non-pharmacological interventions to manage the symptoms of depression in patients undergoing BC therapy. There appears to be good scientific evidence to support the inclusion of yoga and mindfulness practises in standard-of-care interventions to manage depression symptoms in patients with BC. Furthermore, CBT can also be useful in cases with clinical depression, both with a CT approach focusing on moods and thoughts or with behavioural therapy specifically targeting actions and behaviours.

Nonetheless, the implementation of non-pharmacological interventions to manage depression symptoms in clinical practice is still lacking, with most such interventions focusing mainly on the use of antidepressant drugs. These medications have variable response rates that can likely be improved by simultaneously applying non-pharmacological interventions. However, this combined approach is rarely adopted in clinical practice, partly because of the high economic cost of the human resources required to implement these practises. Indeed, in countries with public health systems, these types of interventions are not included in the healthcare services available in hospitals or primary care centres, meaning that patients must access them through non-profit organisations, patient associations, or private clinics.

Increased physical activity may reduce the burden of depression symptoms in patients with BC but the current evidence in this regard is still ambiguous. Because these intervention strategies may differentially impact the symptoms of depression, more work will be required to assess the exercise types, duration, and frequency required to achieve a minimal clinically important difference (MCID) in BC survivors. Considering patient perspectives may also be helpful, given that effect sizes alone cannot indicate the clinical relevance of an intervention because the outcome itself determines this factor.

The MCID is defined as the smallest difference in a domain of interest, without problematic side effects or excessive cost, perceived by patients as beneficial and that would justify a change in their therapeutic management. Thus, this metric could be used as a starting point to pinpoint the cutoff for clinical relevance and in this sense, psychotherapy, pharmacotherapy, and combined treatments have all been shown to have effect sizes above this threshold. Notably, several international oncology societies now recommend exercise in all cancer patients and so its use in the treatment of depression symptoms will likely increase. Thus, more detailed analyses of the efficacy of such programmes to reduce the burden of depression in patients with BC will be warranted to inform the design and optimisation of behavioural interventions for these patients.

Although not overtly considered in the articles included in this review, in our opinion, the use of objective biomarkers measured in biological samples (e.g., blood, saliva, and hair, etc.) could also help to objectively analyse non-pharmacological interventions in terms of psychological alterations. The molecular effects of non-pharmacological interventions have been addressed in few studies focusing on inflammatory cytokines (IL-6, TNF-α, and IL-1β) and the hypothalamic–pituitary–adrenal (HPA) activation hormone, cortisol, and deserve further attention as a biomarker for non-pharmacological interventions as well. The term biomarker refers to any objectively measurable property that is reproducible, reliable, inexpensive, and non-invasive that can serve as a proxy for a normal or pathological biological process or a therapeutic intervention response. Thus, the identification and use of diagnostic biomarkers to distinguish the presence or absence of the symptoms of depression and of treatment biomarkers to predict responses to therapeutic interventions would clearly be beneficial.

Biomarkers are also categorised into trait, state, and endophenotype markers. Trait biomarkers are persistent and highlight biological processes that existed before the onset of the disorder that play a causal role in its pathophysiology. Thus, these biomarkers could be used to determine which individuals are at risk of BC. State biomarkers are transient and related to the clinical condition and so would be present at the onset and during BC but normalise with its remission. Endophenotypic biomarkers are a subgroup of trait biomarkers marking genes involved in certain conditions, and so they are persistent and more common in BC patient family members than in the general population.

As a solution to the problem of the low sensitivity and specificity of single biomarkers, some authors recommend the use of biomarker panels containing several biological factors for the diagnosis of depression and evaluation of its treatment responses. Thus, when trying to measure clinical differences in different therapeutic approaches to treating depression in patients with BC, using a wider range of neuroimaging, genetic, epigenetic, proteomic, and metabolomic biomarkers to incorporate multiple biological abnormalities would likely be beneficial.

## Figures and Tables

**Table 1:** The characteristics of the studies reporting significant effects regarding depressive symptoms after these interventions.

Reference	Number of Participants	Clinical Stage	Treatment	Instrument Used to Evaluate Depression	Depression Severity	Description of the Intervention/Intervention Type	**Results in Terms of the Symptoms of Depression**
(Aydin et al., 2021) [24]	Experimental group: 24 Control group: 24	Did not specify	Had undergone partial or total breast surgery for breast cancer and had not developed metastasis.	Beck Depression Inventory (BDI) [73]	Mild to moderate depression.	The study group received a 12-week aerobic exercise programme at the gym and a home-based resistance exercise program.	This study showed that aerobic and resistance exercises improved and decreased levels of depression.
(Bower et al., 2021) [57]	Experimental groups: MAP: 85 SE: 81 Control group: 81	I, II, III	The patients had received surgery, RT, and chemotherapy 6 months before the intervention.	Depressive symptoms were assessed using the Center for Epidemiological Studies Depression Scale (CES-D). A score of 16 or higher on this scale indicates the presence of clinically significant depressive symptoms [74].	Mild to moderate depression.	The mindful awareness practises (MAP) intervention consisted of theoretical sessions on mindfulness, relaxation, and mind–body connection. The sessions were held for 2 h weekly for 6 weeks. Survivor education (SE) was a standard educational programme that covered important topics, such as quality of life post-BC, medical management after treatment, work–life balance, body image, sexuality, nutrition and physical activity, the impact of cancer in families, and genetics. These sessions were also conducted in groups, with weekly 2 h meetings for 6 weeks.	Reductions in depression symptoms from pre-intervention to post-intervention were observed in both experimental groups compared to the control group.
(Liu et al., 2022) [66]	Experimental group: 68 Control group: 68	I, II	All the patients received surgery and postoperative chemotherapy.	The Hospital Anxiety and Depression Scale was used to assess symptoms of anxiety and depression (HADS), having 10–21 points is indicative of significant symptoms of anxiety or depression [75].	Mild depression.	The experimental group participated in weekly mindfulness yoga sessions that included meditation, breathing-focused yoga poses, and discussions, alongside conventional care for 8 weeks. The control group received routine nursing interventions during the same period, which included psychological care, dietary counselling, health education, telephone follow-ups, and lifestyle counselling.	The experimental group, which practised mindfulness yoga along with conventional mindfulness, significantly improved compared to the control group, especially in terms of depression.
(Ghorbani et al., 2021) [67]	Experimental group: 20 Control group: 20	Did not specify.	Did not specify.	The tool was the Depression, Anxiety and Stress Scale (DASS-21), having a score ≥10 indicates depressive symptoms [76].	Severe depression.	Acceptance and commitment therapy (ACT) sessions were conducted by a psychotherapist and consisted of 8 weekly sessions lasting 90 min each. The control group received no intervention but did receive ACT 4 months later.	Compared to the control group, ACT treatment significantly reduced the mean depression scores and psychological flexibility significantly increased.
(Akkol-Solakoglu and Hevey 2023) [68]	Experimental group: 53 Control group: 23	I–IV	The patients had completed primary treatment and were BC survivors.	The Hospital Anxiety and Depression Scale (HADS) was used to measure anxiety and depression symptoms. A score between 10 and 21 suggests the presence of significant symptoms of anxiety or depression [75].	Mild depression.	The internet-based cognitive behavioural therapy (iCBT) programme was a seven-module, 8-week intervention based on a transdiagnostic approach to depression and anxiety offered through the SilverCloud platform. iCBT in the intervention group aimed to improve the knowledge of BC survivors of their experience and their symptom management skills. The control group continued with the usual care (UC) recommended by their healthcare team.	Although the difference was not significant, the experimental group exhibited lower depression scores compared to the control group after the intervention. This disparity reached statistical significance during the 2-month follow-up.
(He et al., 2022) [69]	Experimental group: 88 Control group: 88	I–III, recently diagnosed.	The patients had received surgery and were scheduled for chemotherapy.	Depression was assessed using the Patient Health Questionnaire Depression Scale (PHQ-9). The total score ranges from 0 (no depression) to 27 (severe depression) [77].	Does not specify.	A Chinese folk ‘square dance’ characterised by its simple steps was adapted for BC patients. The intervention included six instructional sessions in the hospital. The first one lasted 1 h and the others were 30 min, each with short breaks. Additionally, the patients were asked to practice at home for 16 weeks, spending 150 min a week dancing. The control group received a health consultation for 16 weeks as part of the treatment.	Participants who carried out the dance intervention for 16 weeks reported a lower incidence of depression compared to the control group.
(Getu et al., 2023 [70]	Experimental group: 30 Control group: 28	I–IV	The patients were receiving chemotherapy.	Depression assessment was performed using the Patient Health Questionnaire Depression Scale (PHQ-9). The total score ranges from 0, indicating no depression, to 27, indicating severe depression [78].	Does not specify.	The experimental group received seven sessions of cognitive behavioural therapy activity stimulation (CBT-AP) during chemotherapy, with three face-to-face group sessions lasting 2 h and 4 individual telephone sessions lasting 30 min. The control group received UC.	Compared to the control group, the experimental group had lower depression scores.
(Salchow et al., 2021) [27]	Experimental group: 30 Control group: 20	Did not specify.	The patients had completed treatment with surgery, chemotherapy, radiation, and hormone therapy, and had received antibodies 6 months before the intervention.	The level of fear and depression was quantified using the HADS (Hospital Anxiety and Depression Scale), having >10 points is indicative of significant symptoms of anxiety or depression [75].	Mild depression.	The intervention group participated in a Kyusho Jitsu intervention twice a week for 6 months; the control group received no intervention.	There were no significant differences between the groups regarding symptoms of depression, although there was a significant reduction in the anxiety subscale from baseline to 6 months in the intervention group.
(Saxton et al., 2014) [28]	Experimental group: 44 Control group: 41	I–III	The patients had completed surgery, RT, and chemotherapy treatment in the 18 months prior.	Depressive symptoms were assessed using the BDI (Beck Depression Inventory version II: BDI-II) which has a range from 0 to 63 with a cut off ≥14 [73].	Mild depression.	The 24-week lifestyle intervention combined three supervised exercise sessions each week with a personalised, low-calorie healthy eating programme. The exercise sessions comprised 30 min of aerobic exercise.	Compared to the control group, the intervention group showed a reduction in depression symptoms.
(Zhou et al., 2015) [29]	Experimental group: 85 Control group: 85	Did not specify.	The patients were scheduled to undergo a radical mastectomy.	The Zung Depression Self-Rating Scale (ZSDS) was used. The Chinese ZSDS is a 20-item self-report measure that assesses symptoms of depression. The sum of the 20 items produces a score that ranges between 20 and 80, with a higher score demonstrating greater depression [79].	Does not specify.	Two therapeutic techniques were used in the post-mastectomy experimental group: music therapy and muscle relaxation training. Music therapy involved listening to music chosen by the patients twice a day for 30 min (from 6:00 to 8:00 A.M. and 9:00 to 11:00 P.M.) within 48 h of surgery and until hospital discharge. A muscle relaxation training programme with two daily 30 min sessions was simultaneously implemented. The control group received UC.	A significant improvement in depression levels was observed in the experimental group compared to the control group. The implementation of music therapy and progressive muscle relaxation training appeared to reduce both depression and the length of hospitalisation in female patients with BC after radical mastectomy.
(Cheung et al., 2017) [30]	Experimental group: 26 Control group: 13	IV B metastatic BC.	Did not specify.	Depression was measured using the 20-item Center for Epidemiologic Studies Depression Scale (CES-D).	Mild depression.	The LILAC intervention consisted of 5 weekly 1 h sessions in which the participants learned eight evidence-based skills designed to increase the frequency of positive emotions. The participants in the in-person control condition also received individual 5 h sessions. The control sessions consisted of an interview without a didactic intervention or skills practice.	The participants in the experimental group who received a combination of in-person and online therapy exhibited decreased levels of depression and negative affect at the 1-month follow-up.
(Li et al., 2018) [31]	Experimental group: 145 Control group: 136	I–IV	The patients had received conservative surgery and were receiving RT.	The level of anxiety and depression was measured with the Hospital Anxiety and Depression Scale (HADS). A score above 10 indicates high levels of anxiety and depression [75].	Mild depression.	In the intervention group, a 3 h comprehensive educational course on psychological stress and management skills was taught. In the control group, all the patients were offered a basic 15 min educational course on BC and RT on their day of admission, according to the UC guidelines of the hospital.	Both anxiety and depression scores increased during RT. Furthermore, this trial concluded that a comprehensive educational course on BC, with an emphasis on teaching stress management skills, did not lead to significant changes in depression scores over the 5 to 6 weeks of RT.
(Lanctôt et al., 2016) [32]	Experimental group: 58 Control group: 43	I–III	The patients were receiving chemotherapy.	Depressive symptoms were evaluated with the Beck Depression Inventory, version II (BDI-II), whose scores ranges from 0 to 63. The scores are interpreted as follows: 0–10 (normal), 11–16 (mild disturbance), 17–20 (borderline clinical depression), 21–30 (moderate depression), 31–40 (severe depression), and more than 40 (extreme depression) [73].	Mild depression.	The Bali Yoga Programme for BC patients (BYP-BC) consisted of 23 gentle *hatha asanas* (postures), 2 *prayanamas* (breathing techniques), *shavasanas* (‘corpse’ relaxation postures), and psychoeducational topics. The participants attended 8 weekly sessions lasting 90 min each and received a DVD with 20 and 40 min sessions to practice with at home. The participants in the waitlist control group received UC for 8 weeks and then received the same intervention.	The symptoms of depression increased in the control group and no significant changes were recorded in the experimental group. However, a reduction in depression symptoms was observed in the control group after receiving the BYP-BC intervention.
(Lu et al., 2023) [33]	Experimental groups: SR: 46 ESR: 54 CF: 36	0–IV	The patients had completed primary treatment surgery, RT, and chemotherapy within the 5 years prior.	The 10-item Center for Epidemiological Studies Depression Scale. A higher score indicates more depressive symptoms (total score ranged from 0 to 30) [80].	Does not specify	The study divided the participants into three conditions: greater self-regulation (ESR), self-regulation (SR), and facts about cancer (CF). The ESR group wrote about stressful cancer-related experiences and coping strategies in the first week, deep feelings about BC in the second week, and positive thoughts about the experience in the third week. The SR group did the same, but the order of weeks 1 and 2 was changed. The CF group wrote objectively about their diagnosis and treatment.	The ESR group showed improvements in the symptoms of depression, unlike the other two groups, which did not show any significant differences.
(Kiecolt-Glaser et al., 2014) [34]	Experimental group: 100 Control group: 100	0–IIIa	The patients had finished treatment with aromatase inhibitors and were BC survivors.	The Center for Epidemiological Studies Depression Scale (CES-D) assessed depressive symptoms in the last week [74].	Does not specify.	The participants were assigned either to a wait-list control or a group that practised 12 weeks of twice-weekly 90 min hatha yoga classes.	The groups did not differ in terms of depression at any time. Secondary analyses showed that the frequency of yoga practice was strongly associated with fatigue but not with depression.
(Sánchez-Jáuregui et al., 2018) [35]	Experimental groups Hypnosis: 58 Music: 55 Control group: 57	Did not specify.	The patients were scheduled to receive a breast biopsy.	To evaluate anxiety and depression, the HADS scale (Hospital Anxiety and Depression Scale) was used. A score >10 indicates high levels of anxiety and depression [75].	Does not specify.	There were two experimental groups (hypnosis or music therapy) and one control group, with three measurement times: baseline, (the period prior to the intervention) pot-intervention but pre-biopsy, and at the end of the breast biopsy. The hypnosis group received a recorded audio hypnosis intervention. The music group listened to the background music, without the suggestions, for the same period. Patients in the control group spent 17 min in the waiting room.	The results showed a statistically significant reduction in depression in the hypnosis and music groups compared to the control group. Before the biopsy, hypnosis significantly decreased depression levels compared to the music alone, but after the biopsy, there were no differences between the two groups.
(Smith et al., 2019) [36]	Experimental group: 37 Control group: 52	Did not specify.	Did not specify. The patients were BC survivors.	The Patient Health Questionnaire (PHQ-8) was used to capture symptoms of depression. With a cut-off point ≥10 to define current depression [81].	Mild depression.	The Reimagine (symptom self-monitoring) curriculum consisted of web-based content and required activities including attending an online introductory group meeting, viewing videos, and completing mind–body and cognitive reframing exercises over an 18-week period. The programme also taught mind–body exercises such as guided imagery and meditation. The participants in the control group were offered the opportunity to complete Reimagine without cost at the end of the study period.	Reimagine resulted in significant reductions in depression symptoms in the experimental group participants compared to the control group.
(Taso et al., 2014) [37]	Experimental group: 30 Control group: 30	I–III	The patients were receiving chemotherapy at the time of the study.	The mood profile scale was used, which includes 14 items to assess depression and anxiety (seven items each). Items are rated on a 5-point Likert scale (0 to 4), where higher scores indicate higher levels of depression and anxiety [82].	Does not specify.	A 60 min yoga exercise intervention, twice a week for 8 weeks, was implemented in the experimental group during their chemotherapy treatment. The control group received UC.	The 8-week intervention did not significantly improve depression levels in the experimental group.
(Jang et al., 2016) [38]	Experimental group: 12 Control group: 12	0–III	All the patients had completed active treatment with surgery, RT, and chemotherapy at least 2 years before the intervention.	The Personality Assessment Inventory (PAI) was used. PAI is a self-report survey comprising 344 questions. For this study, questions related to depression and anxiety were extracted [83].	Mild depression.	The patients in the mindfulness-based art therapy (MBAT) group received 12 sessions lasting 45 min each. In addition, the Korean psychological intervention of mindfulness-based stress reduction (K-MBSR) was also applied to the mindfulness activities.	Depression decreased significantly in the experimental group. However, there were no significant changes in the control group.
(Chandwani et al., 2014) [39]	Experimental groups Yoga: 53 Stretching: 56 Control group: 54	0–III	The patients were scheduled to undergo RT.	Depression was assessed using the Center for Epidemiological Studies Depression (CES-D) measures [74].	Mild depression.	The participants in the yoga (YG) and stretching (ST) groups attended up to three classes lasting 60 min per week while undergoing 6 weeks of RT.	There were no differences in the symptoms of depression between the two groups.
(Rethorst et al., 2023) [40]	Did not specify.	I–IV	The patients had completed active treatment more than 3 years before registration and were BC survivors.	Depressive symptoms were measured using the Quick Inventory of Depressive Symptomatology (QIDS-SR). The total score ranges between 0 and 27 points [84].	Mild depression.	In the physical activity intervention, the participants were given the goal of completing 150 min of weekly moderate to vigorous physical activity (MVPA) through a combination of supervised in-person sessions and unsupervised sessions. The active life every day (ALED) participants attended 12 bi-weekly educational sessions led by a trained interventionist, which aimed to support behavioural strategies to increase physical activity.	Results indicated that depression symptoms decreased significantly in the entire study sample during the 6-month intervention. The participants who received the ALED intervention experienced greater reductions in the symptoms of depression.
(Wang et al., 2022) [41]	Experimental group: 84 Control group: 84	0–III in newly diagnosed patients.	The patients were scheduled to receive surgery or chemotherapy.	Hospital Anxiety and Depression Scale (HADS). A score >10 indicates high levels of anxiety and depression [75].	Mild depression.	In a group expression intervention implemented during the systemic treatment process, the participants received four sessions of nurse-led support each lasting about 60 min, focussing on the following topics: ‘being patient’, ‘interpersonal relationships’, ‘the journey to recovery’, and ‘planning for the future’. The control group received health education, rehabilitation training, psychological support, and other nursing measures.	The participants in the experimental group reported more significant reductions in depression.
(Ventura et al., 2017) [42]	Experimental group: 105 Control group: 121	I–III	Scheduled for surgery.	Hospital Anxiety and Depression Scale (HADS). A score >10 indicates high levels of anxiety and depression [75].	Does not specify.	An educational intervention with the computer-based Swedish Interactive Rehabilitation Information (SIRI) lectures led by various experts in cancer treatment (surgeons, oncologists, oncology nurses, physiotherapists, social workers, and counsellors), as well as a patient representative. The lectures were presented as Microsoft PowerPoint slides and took a combined time of 4 h.	Multilevel modelling revealed that this educational programme modality had no significant impact on outcomes over time. Compared to the control group, SIRI was not effective in improving patient depression levels.
(Yao et al., 2022) [43]	Experimental group: 36 Control group: 36	I–III A, B, C	The patients had received adjuvant chemotherapy in the 2 months prior.	The Hospital Anxiety and Depression Scale-Depression (HADS-D) was adopted to assess the participant’s depression, where a higher score indicates greater severity of depression [75].	Moderate to severe depression.	All the participants received routine care including an educational pamphlet on self-management of cancer symptoms, while the participants in the Tai Chi group received an additional 8-week Tai Chi intervention with two sessions lasting 60 min per week.	Tai Chi appeared to be effective in relieving the symptoms of depression. The results showed significantly lower scores in the experimental group compared to the control group and baseline.
(Ren et al., 2019) [44]	Experimental groups CBT: 98 SCM: 98 Control group: 196	I–III	The patients had received surgery, RT, chemotherapy, and hormonal therapy.	Depressive symptoms were assessed using the Chinese version of the 17-item Hamilton Depression Rating Scale (HAMD-17). Participants with a HAMD-17 score of ≥8 were considered to have existing depressive symptoms [85].	Mild depression.	There were two groups: (1) psychoeducational cognitive behavioural therapy (CBT) in which the therapist taught behavioural strategies such as meditation and distraction, emotional regulation skills, and social and psychological techniques; and (2) self-care management (SCM), designed to control for the effects of treatment expectations and professional care. Women in the CBT and SCM groups received nine sessions over 12 weeks, while women in the control group received only their UC.	CBT effectively reduced depression symptoms in the experimental group compared to the other two groups. Although the participants in the experimental group who received CBT had higher levels of depression at the beginning of the study, by the end, their scores decreased below the cutoff.
(Belay et al., 2022) [45]	Experimental group: 57 Control group: 57	Patients with and without metastases.	The patients were receiving RT, chemotherapy, and hormone therapy.	Distress was measured using the distress thermometer, a numerical scale from 0 (no distress) to 10 (extreme distress), with a cut-off point of ≥7. The level of depression and anxiety was measured with the Hospital Anxiety and Depression Scale (HADS) with a cut off ≥8 [75].	Moderate depression.	Professional therapists trained in interpersonal psychotherapy administered the Interpersonal Psychotherapy Ethiopian version (IPT-E) intervention involving four to six therapy sessions lasting for 30 to 60 min each week, delivered in person.	IPT-E effectively reduced depression symptoms.
(Mertz et al., 2017) [46]	Experimental group: 25 Control group: 25	Did not specify.	The patients had received surgery, RT, chemotherapy, and hormonal therapy.	Anxiety and depression were measured using the Hospital Anxiety and Depression Scale (HADS) with a cut-off score ≥8 indicating cases of anxiety or depression [75].	Mild depression.	A personalised nursing navigation, which included individual counselling based on manuals incorporating cognitive therapy (CT) and psychoeducation strategies to motivate and support patients in the self-management of their symptoms and use of rehabilitation services. Patients in the control group received UC.	Compared to the control group, women in the intervention group reported significantly lower depression scores after 12 months.
(Ying et al., 2019) [47]	Experimental group: 46 Control group: 40	I–IIIC	The patients had completed active treatment, surgery, chemotherapy, and RT in the 2 years prior and were BC survivors.	The Patient Health Questionnaire (PHQ-9) is a validated nine-item tool to measure the severity of depression, with scores ranging from 0 to 27. Higher scores indicate higher levels of depression [77].	Mild depression.	Over 6 months, the experimental group performed the Baduanjin exercises 3 days a week in the hospital and another 4 days a week at home. The control group was asked to maintain their original daily physical activity for no less than 30 min per day, for 6 months. The patients recorded their daily activities at home.	After completing this specific exercise for 6 months, there were significant improvements in the symptoms of depression in the experimental group compared to the control group.
(Beutel et al., 2014) [48]	Experimental group: 78 Control group: 79	0–III	The patients were recruited after surgery while still undergoing oncological treatment (chemotherapy, radiation, and hormonal treatment).	Anxiety and depression were measured using the Hospital Anxiety and Depression Scale (HADS) with a cut-off score ≥8 indicating cases of anxiety or depression [75]. Depressive disorder according to SCID-I (ICD-10 diagnoses).	Depression clinic.	In the Short-Term Psychodynamic Psychotherapy (STPP) study a treatment manual outlining specific strategies to address serious problems such as hopelessness or suicidal thoughts was used. Up to five preliminary sessions and 20 weekly therapy sessions were offered. The treatment ended when the agreed objectives were achieved. The control group received written information about local cancer counselling centres.	Unlike previous interventions, recruitment to this study required a diagnosis of depression; remission of depression was the primary outcome. Their analyses indicated that approximately twice as many patients (44%) achieved remission after STPP compared to the control group (23%).
(Bower et al., 2015) [49]	Experimental group: 39 Control group: 32	0–III	The patients had completed local and/or adjuvant oncological therapy (except hormonal therapy) at least 3 months previously.	Depression was assessed using the Center for Epidemiological Studies Depression (CES-D) measures [74].	Mild depression.	The intervention was based on the mindful awareness practises (MAP) programme. The participants met for 6 weekly 2 h group sessions that included the presentation of theoretical materials on mindfulness, relaxation, and the mind–body connection.	The MAP intervention produced significant short-term reductions in perceived stress and marginal reductions in depression symptoms.
(Lepore et al., 2014) [50]	Experimental groups: S-ISG: 96 P-ISG: 98	I–II	The patients had received surgery, RT, and chemotherapy.	Anxiety and depression were measured using the Hospital Anxiety and Depression Scale (HADS) with a cut-off score ≥8 indicating cases of anxiety or depression [75].	Mild depression.	There were 2 manualised intervention conditions sharing many characteristics. Both groups had weekly 90 min live (synchronous) chats for 6 weeks. In the Standard Internet Support Groups (S-ISG), peer-to-peer information sharing and emotional support, normalisation of experiences, and promotion of skills and confidence to make positive life changes was emphasised. The Enhanced Prosocial Internet Support Groups (ISG P-ISG) group included all elements of the S-ISG but also received written advice on how to recognise and respond to the online support needs of others.	Relative to the S-ISG, the participants in the P-ISG condition had higher levels of depression and anxiety symptoms following the intervention. The P-ISG did not produce better mental health outcomes in distressed BC survivors compared to the S-ISG.
(Yun et al., 2017) [51]	Experimental group: 22 Control group: 24	I–III	The patients had completed surgery and/or adjuvant chemotherapy and/or RT after surgery up to 2 years prior and 6 months before the study.	Depression was assessed using the Center for Epidemiologic Studies Depression (CES-D) measures, which consisted of 20 items to measure depression symptoms [74].	Mild depression.	The study consisted of a mental subtraction meditation (MSM) programme conducted twice a week for 8 weeks (16 sessions in total). Educational sessions on life after cancer treatment, health exercises, diet management, and follow-up were included. Full-scale meditation began in the fifth session. In comparison, the self-management education (SME) group received the same first 4 sessions as the MSM group, and then received lectures on relationship improvement, communication skills, and stress management.	The experimental group that received the MSM intervention reported a significant decrease in depression, anxiety, and perceived stress, compared to the SME control group.
(Bidstrup et al., 2023) [52]	Experimental group: 156 Control group: 153	Did not specify.	The patients had received surgery, RT, chemotherapy, hormonal therapy, and trastuzumab.	The Patient Health Questionnaire (PHQ-9) is a validated nine-item tool to measure the severity of depression, with scores ranging from 0 to 27. Higher scores indicate higher levels of depression [77].	Moderate depression.	The study implemented the REBECCA nursing navigation intervention. Patients were randomly assigned to this intervention and received counselling and symptom screening by nurses in addition to UC. REBECCA has two components: detection of psychological and physical symptoms and nursing navigation and is delivered in about six individual sessions (the first one in person, and the rest by telephone) lasting approximately 60 min each. UC included regular treatment and nursing support at chemotherapy and RT appointments, as well as rehabilitation.	The nursing navigation and symptom screening provided in the REBECCA intervention showed promise in reducing several psychological symptoms. Significant effects were observed for depression symptoms at the 6-month follow-up.
(Bredal et al., 2014) [53]	Experimental groups: SG: 182 PEG: 185	I–III	The patients had received surgery, RT, chemotherapy, and hormonal therapy.	Anxiety and depression were measured using the Hospital Anxiety and Depression Scale (HADS) with a cut-off score ≥8 indicating cases of anxiety or depression [75].	Mild depression.	Psychoeducational groups (PEG) and support groups (SG) were compared. The PEG intervention consisted of 5 weekly 2 h sessions addressing health education, stress management, problem-solving skills, and psychological support. The SG received 3 weekly 2-h sessions. Both interventions sought to reduce the feeling of alienation and isolation by creating an environment of acceptance and providing support, information, and dispelling misperceptions and fears about adjuvant treatment.	Regarding depression, both the PEG and SG interventions produce improvements over time; However, there were no significant differences between the groups.
(Kim et al., 2018) [54]	Experimental group: 30 Control group: 30	I–III	The patients were receiving chemotherapy.	Anxiety and depression were measured using the Hospital Anxiety and Depression Scale (HADS) with a cut-off score ≥8 indicating cases of anxiety or depression [75].	Mild depression.	The nurse-led psychological intervention programme comprised 7 weekly counselling sessions delivered face-to-face and by telephone. The programme provided education on chemotherapy symptom management and skills for coping with negative emotions during BC treatment.	Compared to the control group, the intervention group reported significantly fewer symptoms of depression.
(Badger et al., 2020) [55]	Experimental groups: SHE: 114 TIPC: 116	I–IV	The patients had finished active treatment a year prior and were BC survivors.	Depression and anxiety were assessed using eight-item short forms from the Patient-Reported Outcomes Measurement Information System (PROMIS). Higher scores equal more of the mastery being measured [86].	Mild depression.	The telephone interpersonal counselling (TIPC) intervention focused on mood management, emotional expression, communication, interpersonal relationships, social support, and cancer information, with sessions lasting approximately 30 min for survivors and 29 min for caregivers. Supportive health education (SHE) focused on breast health, routine testing, treatment, side effects, and coping strategies, as well as lifestyle interventions such as nutrition and physical activity, with sessions lasting around 27 min for survivors and 24 min for caregivers.	The TIPC intervention produced lower depression scores compared to the SHE.
(Nissen et al., 2020) [56]	Experimental group: 82 Control group: 46	Did not specify.	The patients had completed primary treatment for breast or prostate cancer and were cancer survivors.	Beck Depression Inventory (BDI-II). The lowest possible score is 0 and the highest possible score is 63. Higher scores indicate the presence of depression [73].	Mild depression.	An internet-delivered mindfulness-based CT (iMBCT) programme was developed in collaboration with representatives of cancer survivors. There were eight modules lasting 1 week. which included written material, audio exercises, writing tasks, specific cancer patient examples, and videos with patients and experts.	In the experimental group, depression outcomes improved immediately after the intervention.
(Shao et al., 2021) [58]	Experimental group: 72 Control group: 72	I–IV	The patients were undergoing surgery and had received chemotherapy and RT.	The Patient Health Questionnaire (PHQ-9) is a validated nine-item tool to measure the severity of depression, with scores ranging from 0 to 27. Higher scores indicate higher levels of depression [77].	Mild depression.	Participants in the experimental group received guided self-help rooted in mindfulness-based stress reduction practises and CT. The patients were instructed to follow certain daily practices at home for 6 weeks, while the waiting list control group received UC with health education and follow-ups.	There were significant improvements in depression symptoms in the experimental group compared to waiting list controls, and these improvements were maintained at the 1- and 3-month follow-ups.
(Raji Lahiji et al., 2022) [59]	Experimental group: 46 Control group: 44	0–III C	The patients had completed active treatments at least 3 months before the study enrolment.	Beck Depression Inventory (BDI-II). The lowest possible score is 0 and the highest possible score is 63. Higher scores indicate the presence of depression [73].	Mild depression.	Over 2 months, the participants received four nutritional counselling sessions covering diet education, depression prevention and mood improvement recommendations, lasting 120 min. In addition to nutritional counselling, the participants in the combined intervention group attended weekly psychotherapy sessions based on logotherapy principles with the same delivery pattern.	After 8 weeks, the participants in speech therapy with nutrition education classes reported significantly lower depression scores compared to those participating in nutrition education classes alone.
(Desautels et al., 2018) [60]	Experimental groups: CT: 25 BLT: 26 Control group: 11	0–III	The patients had received active treatment: surgery, RT, hormone therapy, and chemotherapy.	The Hospital Anxiety and Depression Scale (HADS-D) [75], the Beck Depression Inventory-II (BDI-II) [73] and the Hamilton Depression Rating Scale (HDRS) [87].	Mild depression.	The cognitive therapy (CT) group received 8 weekly sessions of approximately 60 min. The bright light therapy (BLT) participants were instructed to expose themselves to a 10,000-lux light box for 30 min every morning, before 10 a.m. The control group were contacted every 2 weeks to investigate the presentation of depression symptoms or suicidal ideation.	After treatment, there was a significant reduction in the symptoms of depression in patients who received CT compared to the CG, as measured by two different evaluation scales. The BLT participants showed a greater decrease in depression symptoms only on one scale when compared to the CG.
(Boing et al., 2018) [61]	Experimental group: 8 Control group: 11	Did not specify.	At the time of the study, the patients were receiving hormonal therapy, chemotherapy, and RT.	Beck Depression Inventory (BDI-II). The lowest possible score is 0 and the highest possible score is 63. Higher scores indicate the presence of depression [73].	Mild depression.	The experimental group took 12 weeks of belly dance classes lasting 60 min each, twice a week.	Belly dancing significantly reduced the symptoms of depression in women with BC.
(Blanco et al., 2019) [62]	Experimental groups: IPT: 46 PST: 43 BSP: 45	I–IV	Did not specify.	Beck Depression Inventory (BDI-II). The lowest possible score is 0 and the highest possible score is 63. Higher scores indicate the presence of depression [73].	Major depressive disorder.	There were three interventions lasting 45 min, delivered weekly for 12 sessions. Interpersonal psychotherapy (ITP) helps patients resolve a crisis in their functional or social environment, leading to an improvement in the symptoms of depression. Problem solving therapy (PST) is a cognitive behavioural intervention. The goal is to encourage the adoption and implementation of adaptive problem-solving attitudes and behaviours. Brief supportive psychotherapy (BSP) is an active treatment that uses techniques such as clarification, suggestions, praise, reassurance, normalisation, rehearsal, and anticipation to promote a supportive relationship between the patient and therapist.	No statistically significant differences were observed between the treatment groups, with all three brief psychotherapies showing similar improvements in depression symptoms, with large pre-post effect sizes.
(Rogers et al., 2017) [63]	Experimental group: 110 Control group: 112	Ductal carcinoma in situ or stage I–IIIA BC.	The patients had completed the primary treatment of surgery, RT, chemotherapy, and hormonal therapy less than 1 year prior and were BC survivors.	The 14-item Hospital Anxiety and Depression Scale (HADS) measured depression and anxiety symptomatology, with a cutoff score ≥8 indicating cases of anxiety or depression [75].	Does not specify.	The 3-month social cognitive theory-based BEAT Cancer intervention included 12 supervised exercise sessions. The UC participants received publicly available printed materials from the American Cancer Society but were given no advice or instructions related to physical activity beyond these materials.	Compared with the control group, the symptoms of depression significantly improved in the BEAT Cancer intervention experimental group.
(Carter et al., 2018) [64]	Experimental group: 16 Control group: 11	Ductal carcinoma in situ or stage I–IIIA BC.	The patients had not received chemotherapy or RT during the study period.	Self-reported depression was assessed using the Hospital Anxiety and Depression Scale (HADS) in which higher scores corresponded to greater depression [75].	Does not specify.	Individuals randomised to the physical activity behaviour change intervention (INT) completed 12 supervised exercise sessions. The exercise volume was increased so that during the final 4 weeks of the intervention, the participants performed at least 150 min of moderate-intensity exercise per week. Individuals randomly assigned to the control group received materials available from the American Cancer Society outlining recommendations for exercise and physical activity but no additional contact other than regular telephone check-ins with research staff.	Depression symptoms decreased from the baseline to the follow-up in the experimental group.
(Zahedian et al., 2021) [65]	Experimental group: 12 Control group: 12	Did not specify.	The patients were receiving chemotherapy.	Beck Depression Inventory (BDI-II). The lowest possible score is 0 and the highest possible score is 63. Higher scores indicate the presence of depression [73].	Mild to moderate depression.	Eight 90 min metacognitive therapy (MCT) sessions were delivered to the intervention group. Each week there was a group session in which tasks during and between sessions were set. The control group received UC for depression (except psychotherapy).	The mean depression score in the experimental group had reduced at the 1-month follow-up and there were no significant differences in the control group.
(Stagl, Antoni et al., 2015) [71]	Experimental group: 120 Control group: 120	0–IIIB	The patients had completed active treatments, surgery, RT, chemotherapy, and hormonal therapy, and were BC survivors of 5 years.	Depressive symptoms were assessed using the Center for Epidemiological Studies Depression Scale (CES-D) with a cut off ≥16 [74].	Does not specify.	The cognitive behavioural stress management (CBSM) intervention consisted of a 10-week group programme for women undergoing treatment for BC that combined CBT and relaxation techniques to reduce stress and negative mood. The control group received a 1-day psychoeducational seminar that provided information about BC but did not include structured stress management practises.	Women in the experimental group who received the CBSM intervention reported fewer depression symptoms than women in the control group at the 5-year follow-up. Psychosocial interventions in the early stages of treatment may influence the long-term psychological well-being of BC survivors.
(Stagl, Bouchard et al., 2015) [72]	Experimental group: 51 Control group: 49	0–IIIB	The patients had received surgery, chemotherapy, RT, and hormone therapy 8 to 15 years after enrolment in the study.	Depressive symptoms were assessed using the Center for Epidemiological Studies Depression Scale (CES-D) with a cut off ≥16 [74].	Does not specify.	A 10-week CBSM intervention group programme for women undergoing treatment for BC that combined CBT and relaxation techniques to reduce stress and negative mood was implemented. The control group received a 1-day psychoeducational seminar that provided information about BC but did not include structured stress management practises.	The participants assigned to the CBSM reported significantly fewer symptoms of depression and those that received CBSM after surgery for early-stage BC reported lower depression symptoms than the control group up to 15 years later.

Abbreviations: mindful awareness practises (MAP); cognitive therapy (CT); survivor education (SE); waiting list control (WLC); self-regulation (SR); greater self-regulation (ESR); facts about cancer (CF); cognitive behavioural therapy (CBT); self-care management (SCM); usual care (UC); supportive health education (SHE); telephone interpersonal counselling (TIPC); interpersonal psychotherapy (ITP); problem solving therapy (PST); brief supportive psychotherapy (BSP); intervention to improve exercise adherence after cancer treatment (BEAT Cancer); intervention for physical activity behaviour change (INT); metacognitive therapy (MCT); acceptance and commitment therapy (ACT); internet-based cognitive behavioural therapy (iCBT); cognitive behavioural therapy-activity stimulation (CBT-AP); lessons for linking affect and coping (LILAC); breast cancer (BC), radiotherapy (RT); Bali Yoga Programme for Breast Cancer Patients (BYP-BC); mindfulness-based art therapy (MBAT); Korean mindfulness-based stress reduction (K-MBSR); yoga (YG); moderate to vigorous physical activity (MVPA); active life every day (ALED); Swedish Interactive Rehabilitation Information (SIRI); interpersonal psychotherapy Ethiopian version (IPT-E); standard internet support group (S-ISG); enhanced prosocial internet support group (ISG P-ISG); mental subtraction meditation (MSM); self-management education (SME); psychoeducational group (PEG); support group (SG); bright light therapy (BLT); cognitive behavioural stress management (CBSM); internet-based mindfulness-based cognitive therapy (iMBCT).

## Data Availability

Not applicable.
